# Comparison of the efficacies of 1.0 and 1.5 mm silicone tubes for the treatment of nasolacrimal duct obstruction

**DOI:** 10.1038/s41598-022-16018-4

**Published:** 2022-07-11

**Authors:** Jutaro Nakamura, Tomoyuki Kamao, Arisa Mitani, Nobuhisa Mizuki, Atsushi Shiraishi

**Affiliations:** 1 Department of Ophthalmology, Ehime University Graduate School of Medicine, Shitsukawa, Toon, Ehime 791-0295 Japan; 2 Department of Ophthalmology and Visual Science, Yokohama City University Graduate School of Medicine, Yokohama, Kanagawa 236-0004 Japan

**Keywords:** Eye diseases, Lacrimal apparatus diseases, Outcomes research, Clinical trials

## Abstract

This retrospective observational study analyzed the postoperative outcomes of bicanalicular intubation using different diameters of tube stents for treating postsaccal nasolacrimal duct obstruction. A total of 130 patients diagnosed with postsaccal obstruction who underwent endoscopic-assisted silicone tube intubation were included in the study. Patients intubated with a 1.5-mm large-diameter tube were designated as the LD group, and those with a 1.0-mm normal-diameter tube were designated as the ND group. The patency rates of the two groups at 1 year after tube removal were compared using the Kaplan–Meier curve and restricted mean survival time (RMST) method with τ = 365 days. Results demonstrated that the recurrence rate after tube removal was significantly lower in the LD group as compared with the ND group (*p* = 0.001). The patency rates at 1 year after removal in the LD and ND group were 85.7% (95% confidence interval [CI]: 75.4, 91.9) and 73.9% (95% CI: 61.7, 82.8), respectively. When comparing the patency rates by the RMST method at τ = 365 days, the RMST difference, RMST ratio, and RMTL ratio were higher in the LD group at *p* = 0.045, 0.052, and 0.046, respectively.

## Introduction

Primary acquired nasolacrimal duct obstruction (PANDO) is an organic obstruction of the lacrimal tract that can occur anywhere from the punctum to the opening of the nasolacrimal duct^1^. *Presaccal obstruction* refers to an obstruction from the punctum to the internal common punctum, whereas *postsaccal obstruction* can be found from the lacrimal sac to the nasolacrimal duct opening. Although the pathophysiology of PANDO remains unclear, it is suggested that descending inflammation from the eyes or ascending inflammation from the nose triggers swelling of the mucosal membrane, remodeling of the connective tissue, and subepithelial cavernous body dysfunction with reactive hyperemia, resulting in temporary obstruction of the lacrimal duct^2^. Furthermore, chronic recurrent inflammation in the lacrimal duct causes structural alterations in the epithelium and subepithelial tissue, resulting in the fibrous organic obstruction of the lumen.

First-line treatment for postsaccal obstruction in adults is bypass surgery, namely, dacryocystorhinostomy (DCR). Meanwhile, with the development of the dacryoendoscope and advances in the fiber-optic system, recanalization procedures have steadily evolved. Lacrimal recanalization surgery includes endoscopic-guided trephination DCR, dacryorhinotomy, microdrill dacryoplasty, laser dacryoplasty, and anterograde balloon dacryoplasty^3–10^. Silicone tube intubation and anterograde balloon dacryoplasty are commonly used for treating partial obstruction or stenosis of the lacrimal duct^11–13^. In Northeast Asia, endoscopic-assisted nasolacrimal duct intubation (ENDI) has been proposed as an alternative to DCR for the treatment of postsaccal obstruction. This is primarily because of improved postoperative outcomes with refined tube stents and an improved visibility and accuracy of dacryoendoscopes^14,15^. The ENDI procedure is conducted while directly observing the obstructed site in the lacrimal duct using a dacryoendoscope and observing the nasal cavity using a nasal endoscope (Supplementary file, Video 1). As compared with conventional blind direct silicone intubation, ENDI reduces complications from false passage formation. This procedure is typically performed under local anesthesia, and it has evolved into a less-invasive and more secure procedure, resulting in its increasingly widespread use in Northeast Asia^16–19^. Furthermore, as compared with other ethnical populations, Northeast Asians have relatively flat facial features, with a less elevated superior orbital rim, allowing for relatively easy manipulation of the dacryoendoscope^20^. Since the first report of nasolacrimal duct intubation using silicon tubes by Gibbs et al. and Keith et al. in the 1960^21,22^, various improvements have been made in terms of surgical techniques, instruments for stent placement, tube materials, and designs^4,23^. The insertion of tube stents into the lacrimal duct prevents the adhesion of the mucosal surface of the duct while the mucosal membrane heals, promoting regeneration of the lacrimal duct epithelium and helping to maintain long-term patency after tube removal. Typical tube stents include the Ritleng lacrimal intubation set and the Crawford tube (FCI Ophthalmics, Pembroke, MA), which have been used in many institutions worldwide. Nevertheless, these tubes require a thin metal probe for insertion, which carries a risk of iatrogenic trauma to the canaliculi and nasolacrimal ducts^23^.

The Nunchaku-style tube (NST) is a “push-style” stent designed with a metal guide probe concealed inside the tube^24^. In addition, because it does not require stent retrieval or tying of the distal end of the tubes within the nasal cavity during placement, the NST has improved surgical efficacy^24,25^. The diameter of a typical NST is 1.0 mm, which is thicker than that of the Crawford tube, which has a diameter of 0.64 mm. It has been reported that intubation of large-caliber tubes helps maintain a wide lumen^26,27^. Recently, a thicker 1.5-mm diameter NST has been introduced and is currently available for clinical use. Accordingly, this study was designed to compare the efficacy of 1.0-mm and 1.5-mm NST for the ENDI treatment of postsaccal obstruction.

## Methods

### Subjects

**Figure 1:**
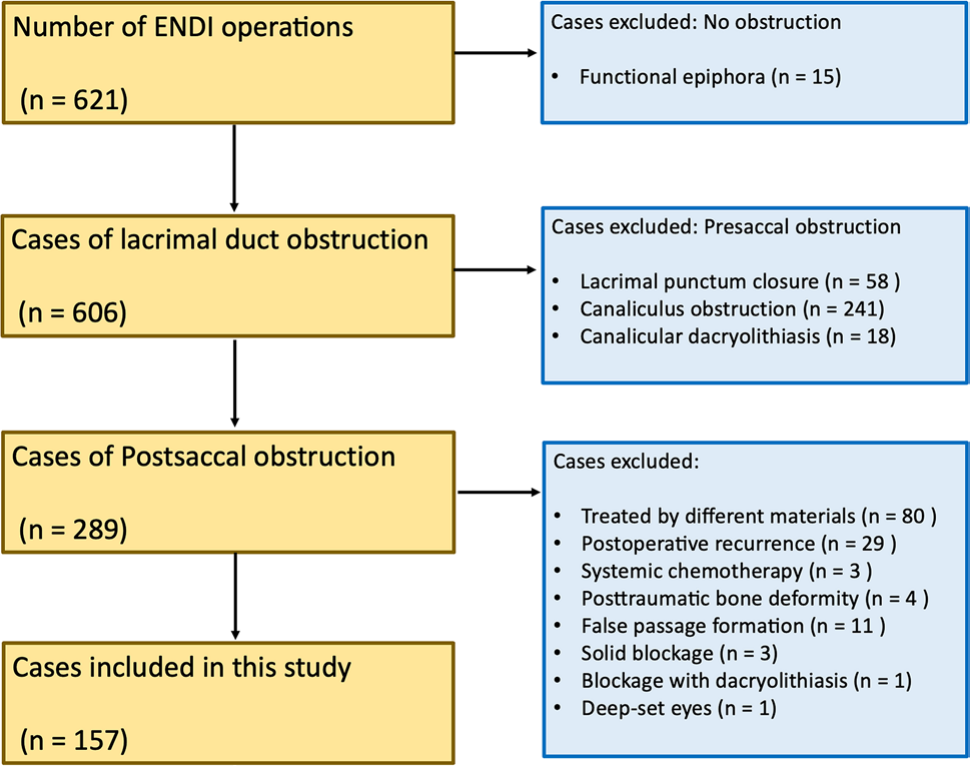
Flow diagram for the included cases in this study. Among the 621 ENDI operations, 15 cases had no obstruction, 317 cases had presaccal obstruction, and 289 cases had postsaccal obstruction. Of the 289 ENDI operations performed for postsaccal obstruction, we identified 16 cases of surgical failure. Of these, 11 were due to false passage formation, 3 were due to solid blockage, 1 was a blockage due to dacryolithiasis, and 1 was due to deep-set eyes. Abbreviation: ENDI, endoscopic-assisted nasolacrimal duct intubation.

We retrospectively investigated 157 sides of 130 patients diagnosed with postsaccal obstruction. Patients were treated at Ehime University Hospital between August 2013 and November 2020 by three ophthalmologists (TK, AM, and AS) with 10, 4, and 14 years of ENDI surgical experience, respectively. The mean age of the patients was 72.6 years, with a standard deviation (*SD*) of 11.3 years. Forty patients (50 sides) were male and 90 (107 sides) were female. Postsaccal obstruction was diagnosed based on dye disappearance tests, lacrimal irrigation test, cone-beam computed tomography digital subtraction dacryocystography (CT-DCG), and dacryoendoscopic examinations. We excluded patients with functional nasolacrimal duct obstruction from the statistical analysis. Patients who had a history of systemic chemotherapy with fluorouracil and/or the taxanes, radiation therapy, or posttraumatic bone deformity were also excluded. In addition, we excluded patients with unsuccessful surgery, such as cases in which the stent could not be placed due to an occlusion that was too solid or cases in which a false passage was created, from the assessment. Patients with a history of lacrimal duct reconstruction (i.e., postoperative recurrence) were also excluded; hence, all patients were treated for the first time (Fig. 1).

### Treatment protocols

**Figure 2:**
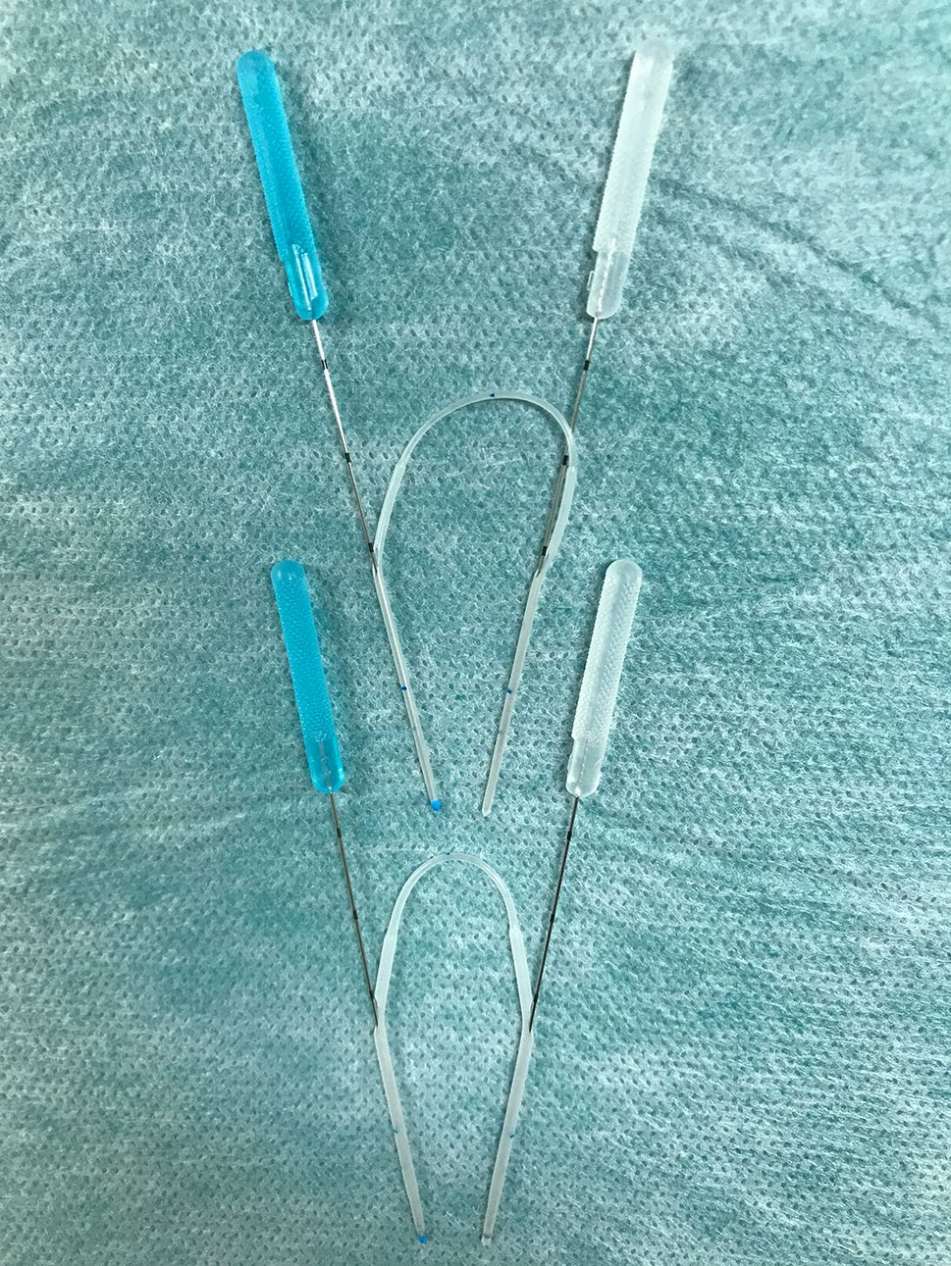
Two types of different-thicknesses NSTs. Shown at the top is a 1.0-mm-diameter tube, and the bottom is a 1.5-mm-diameter tube. The NST is designed with a metal guide probe concealed in the lumen of the tube. Abbreviation: NST, Nunchaku-style tube.

All patients were treated with ENDI as previously described^17,28^. In this procedure, the surgeons performed lacrimal duct reconstruction while observing the lacrimal duct under the dacryoendoscope instead of performing the procedure blindly. Briefly, the procedure was performed as follows: after applying infratrochlear anesthesia and topical lacrimal duct and nasal mucosal anesthesia, the upper and lower lacrimal punctum were dilated. Dacryoendoscope (FT-201, Fibertech, Tokyo, Japan) was inserted through the punctum, and the endoscope was then advanced to the occlusion site while monitoring the lumen. An 18-gage catheter (SR-FF1864, Terumo, Tokyo, Japan) attached to the tip of the endoscope was used to release the obstruction (sheath-guided endoscopic probing technique)^29^. Self-retaining bicanalicular lacrimal stents were inserted using the catheter as a guide after the obstructed area of the lacrimal canal was released. The catheter with the tube connected was removed from the open end of the nasolacrimal duct on the inferior meatus under the view of a nasal endoscope. The same procedure was performed on the other punctum, and the tube was placed in the lacrimal duct (Supplementary file, Video 1). The NST stent used was either a large-diameter 1.5 mm (Lacrifast EX; Kaneka Co., Ltd., Osaka, Japan) or a conventional diameter 1.0 mm (Lacrifast or Lacrifast CL; Kaneka Co., Ltd.; Fig. 2). The only difference between the 1.0-mm-diameter Lacrifast and Lacrifast CL was whether the tube stent had an open end or a blind end, and the rest of the components, including the tube diameter, total length, and material, were identical. Since the deployment of the 1.5-mm NST in August 2017, we have adopted an indication of intubating 1.5-mm NST for all postsaccal obstruction patients.

All patients were treated with topical 0.1% fluorometholone and 0.3% gatifloxiacin four times a day after ENDI surgery. The lacrimal ducts were flushed by saline irrigation periodically until the stent was removed. The tube stent was principally removed 10 to 12 weeks after ENDI surgery.

### Postoperative outcome assessments

We evaluated patients who were eligible for follow-up for at least 6 months after tube removal. The present study was designed to assess the patency rate of postsaccal obstruction after ENDI treatment; hence, we excluded cases of presaccal obstruction. Recurrence was defined as follows. After the tube removal, recurrence was represented as no passage or pus or viscous fluid reflux in the postoperative irrigation test with structural reobstruction in the dacryoendoscopic findings. Meanwhile, during tube placement, recurrence was regarded as no passage with pus or viscous fluid reflux in the irrigation test accompanying granulomatous reocclusions in the dacryoendoscopic findings, requiring further treatments such as tube replacement or DCR surgery. *Functional success* was defined as a reduction in patients’ epiphora and discharge symptoms caused by lacrimal duct obstruction. The success rate was assessed based on a questionnaire routinely administrated 6 months after tube removal.

**Figure 3:**
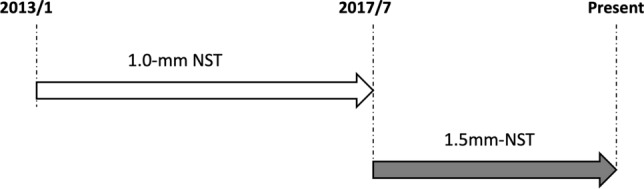
Composition of the ND and LD groups. The ND and LD groups are indicated by the white gray arrows, respectively. The ND group consisted of patients treated with the 1.0-mm NST before the deployment of the 1.5-mm NST (treated between August 2013 and July 2017). Meanwhile, the LD group consisted of patients treated after the deployment of the 1.5-mm NST at our institution (treated between August 2017 and November 2020). Abbreviation: NST, Nunchaku-style tube.

The group intubated with a large-diameter tube (1.5 mm) was designated as the LD group, and the group intubated with a normal-diameter tube (1.0 mm) was designated as the ND group. The LD group consisted of patients treated (between August 2017 and November 2020) after the deployment of the 1.5-mm NST at our institution, and the ND group consisted of patients treated (between August 2013 and July 2017) with the 1.0-mm NST before the deployment of the 1.5-mm NST (Fig. 3, Supplementary Fig. 1). Gender, age, period of obstruction, duration of tube intubation, observation period after removal, number of recurrences, and time from tube removal to recurrence for each group were evaluated. With regard to the time from tube removal to recurrence, the observation period after removal was regarded as zero in case of recurrence while the tube was still in placement. The period of obstruction was denoted based on the duration of chronic epiphora symptoms as described in the patient questionnaire. By defining “recurrence” as an event, we created a Kaplan–Meier curve. Based on the rationale listed below, the restricted mean survival time (RMST) was compared between the two groups with τ = 365 days. (1) Clinically, recurrence is usually observed within 1 year after removal. (2) In our institution, postoperative follow-up is usually completed when no recurrence is observed for 1 year after removal; accordingly, the censoring rate increases beyond 1 year after removal.

### Statistical analysis

All statistical analyses were performed with EZR (Saitama Medical Center, Jichi Medical University, Saitama, Japan), a graphical user interface for R (The R Foundation for Statistical Computing, Vienna, Austria)^30^. In comparing the two groups, the ratio of gender, surgery success rate, and number of recurrences were tested using Pearson χ^2^ test. Meanwhile, age, period of obstruction, duration of tube intubation, and observation period after tube removal were tested by Mann–Whitney *U* test. Using the R software survRM2 package, we analyzed the creation of the Kaplan–Meier curve and comparison of survival rates between the two groups using the RMST method^31,32^. A *p* value of < 0.05 was considered statistically significant.

### Ethical approval and consent to participate

This study and the data collection protocol were authorized by the institutional review board of Ehime University (ethical approval No. 1601003). The research was recorded with the University Hospital Medical Information Network Clinical Trials Registry (UMIN 000,025,180). Each patient provided documented informed consent before registration. All procedures used in this study were conducted following the principles of the Declaration of Helsinki.

## Results

Among the 621 ENDI operations performed at Ehime University Hospital between August 2013 and November 2020, we identified 15 cases with no obstruction, 317 cases with presaccal obstruction, and 289 cases with postsaccal obstruction. Of the 289 ENDI operations for postsaccal obstruction, 16 cases (5.5%) of surgical failure were identified. Of these, 11 were due to false passage formation, 3 were due to solid blockage, 1 was due to a blockage with large dacryolithiasis, and 1 was due to deep-set eyes (Fig. 1). Table 1 presents an overview of the LD and ND groups. The composition of the LD group was as follows. Of the 70 cases and 85 sides (right 47, left 38) with postsaccal obstruction, males comprised 23 cases and 29 sides (32.8% and 34.1%, respectively), whereas females comprised 47 cases and 56 sides (67.1% and 65.9%, respectively). The mean age of the cases was 72.5 years (*SD* = 11.3). The mean period of preoperative obstruction was 29.6 months (*SD* = 40.0). The mean duration of tube intubation was 88.9 days (*SD* = 23.7). The mean observation period after tube removal was 9.8 months (*SD* = 5.9). In the LD group, recurrence after ENDI treatment occurred in 12 cases (14.0%). Among these, four cases experienced recurrence during tube placement (33.3%). The average time from tube removal to recurrence was 3.4 months (*SD* = 4.6).

**Table 1:** Overview of the LD and ND groups.

	Group		*p*-value
	LD	ND	
Tube diameter	1.5 mm	1.0 mm	
Number of cases	N = 70	N = 60	
Number of sides	n = 85 (R 47, L 38)	n = 72 (R 32, L 40)	0.18
Age (years)	72.5 ± 11.1	72.9 ± 11.5	0.73
Gender (male/female)	23 / 47	17 / 43	0.58
Period of preoperative obstruction (months)	29.6 ± 40.0	63.0 ± 91.8	0.009
Duration of tube intubation (days)	88.9 ± 23.7	112.0 ± 53.5	0.036
Observation period after tube removal (months)	9.8 ± 5.9	14.0 ± 14.7	0.38
Functional success rate	72 (84.7%)	41 (56.9%)	0.001
Total number of recurrences	12 (14.0%)	27 (37.5%)	0.001
Number of recurrences during tube placement	4 (4.7%)	14 (19.4%)	0.004
Number of recurrences after tube removal	8 (9.4%)	13 (18.1%)	0.11
< 6 months after removal	4 (4.7%)	2 (2.8%)	0.53
< 1 year after removal	6 (7.1%)	4 (5.6%)	0.70
< 2 years after removal	8 (9.4%)	9 (12.5%)	0.54
> 2 years after removal	0 (0%)	4 (5.6%)	0.028
Time from tube removal to recurrence (months)	3.4 ± 4.6	11.0 ± 16.0	0.82

Data are presented as mean ± standard deviation for age, period of preoperative obstruction, duration of tube intubation, and observation period after tube removal. A *p* value of < 0.05 was considered statistically significant.

On the other hand, the composition of the ND group was as follows. Of the 60 cases and 72 sides (right 32, left 40) with postsaccal obstruction, males comprised 17 cases and 21 sides (28.3% and 29.2%, respectively), whereas females comprised 43 cases and 51 sides (71.7% and 70.8%, respectively). The mean age of the patients was 72.9 years (*SD* = 11.5). The mean period of preoperative obstruction was 63.0 months (*SD* = 91.8). The mean duration of tube intubation was 112.0 days (*SD* = 53.5). The mean observation period after tube removal was 14.0 months (*SD* = 14.7). In the ND group, recurrence after ENDI treatment occurred in 27 cases (37.5%), and 13 of these cases experienced recurrence during the tube placement (48.1%). The average time from tube removal to recurrence was 11.0 months (*SD* = 16.0).

No statistically significant differences were found between the LD and ND groups in age, gender ratio, or observation period after tube removal (*p* > 0.05). Meanwhile, the period of preoperative obstruction and duration of tube intubation were significantly longer in the ND group (*p* = 0.009 and 0.036, respectively, Supplementary Figs. 2 and 3). After ENDI, the number of recurrences was significantly lower in the LD group than in the ND group (*p* = 0.001). Among them, the number of recurrences during tube placement was significantly smaller in the LD group than that of the ND group (*p* = 0.004). The time to recurrence after tube removal was 3.4 months (*SD* = 4.6) in the LD group and 11.0 months (*SD* = 16.0) in the ND group, with no significant difference between the two groups (*p* = 0.82). The functional success was observed in 72 cases (84.7%) in the LD group and 41cases (56.9%) in the ND group, with a significantly higher rate in the LD group (*p* = 0.0001).

As mentioned in the Method section, Lacrifast has an open end and Lacrifast CL has a blind end, although with the same 1.0-mm thickness. We then examined the difference in recurrence rates between the open and the blind ends of the same caliber in the ND group. Eighteen of 53 patients who underwent insertion of open end Lacrifast experienced a recurrence (34.0%), whereas 9 of 19 patients had the blind end Lacrifast CL inserted had a recurrence (47.4%); accordingly, there was no significant difference in the recurrence rates between the open and the blind ends of the same caliber (*p* = 0.30).

Next, since the preoperative obstruction period and the duration of tube intubation were significantly longer in the ND group than in the LD group, we conducted a logistic regression analysis with “period of preoperative obstruction,” “duration of tube intubation,” and “group” as explanatory variables and “recurrence” as the objective variable to examine whether these factors contributed to the recurrence. The results showed that the odds ratio (OR) for “period of preoperative obstruction” was 0.997 (95% confidence interval [CI]: 0.992–1.000, *p* = 0.355), the OR for “duration of tube intubation” was 1.020 (95% CI: 1.000–1.030, *p* = 0.006), and the OR for “group” was 2.92 (95% CI: 1.28–6.69, *p* = 0.011). Hence, “group” and “duration of tube intubation” were significantly correlated with “recurrence”; meanwhile, the “period of preoperative obstruction” was not significantly correlated in this cohort.

**Figure 4:**
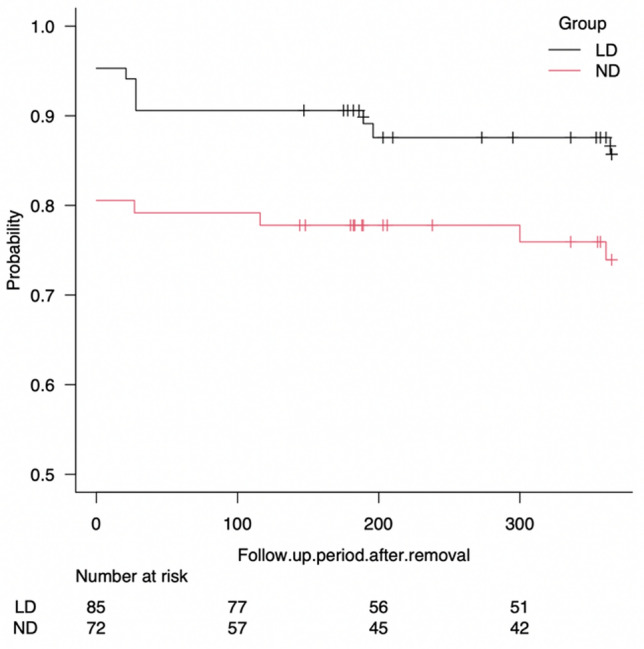
Kaplan–Meier curve of lacrimal duct patency at 1 year after tube removal. The Kaplan–Meier curve indicates that the lacrimal duct patency rate was 85.7% (95% CI; 75.4, 91.9) in the LD group and 73.9% (95% CI; 61.7, 82.8) in the ND group at 1 year after the tube removal. There were 11 recurrences and 23 censored cases in the LD group and 18 recurrences and 14 censorings in the ND group. The *y*-axis denotes the patency rate, and the *x*-axis indicates the number of days.

**Figure 5:**
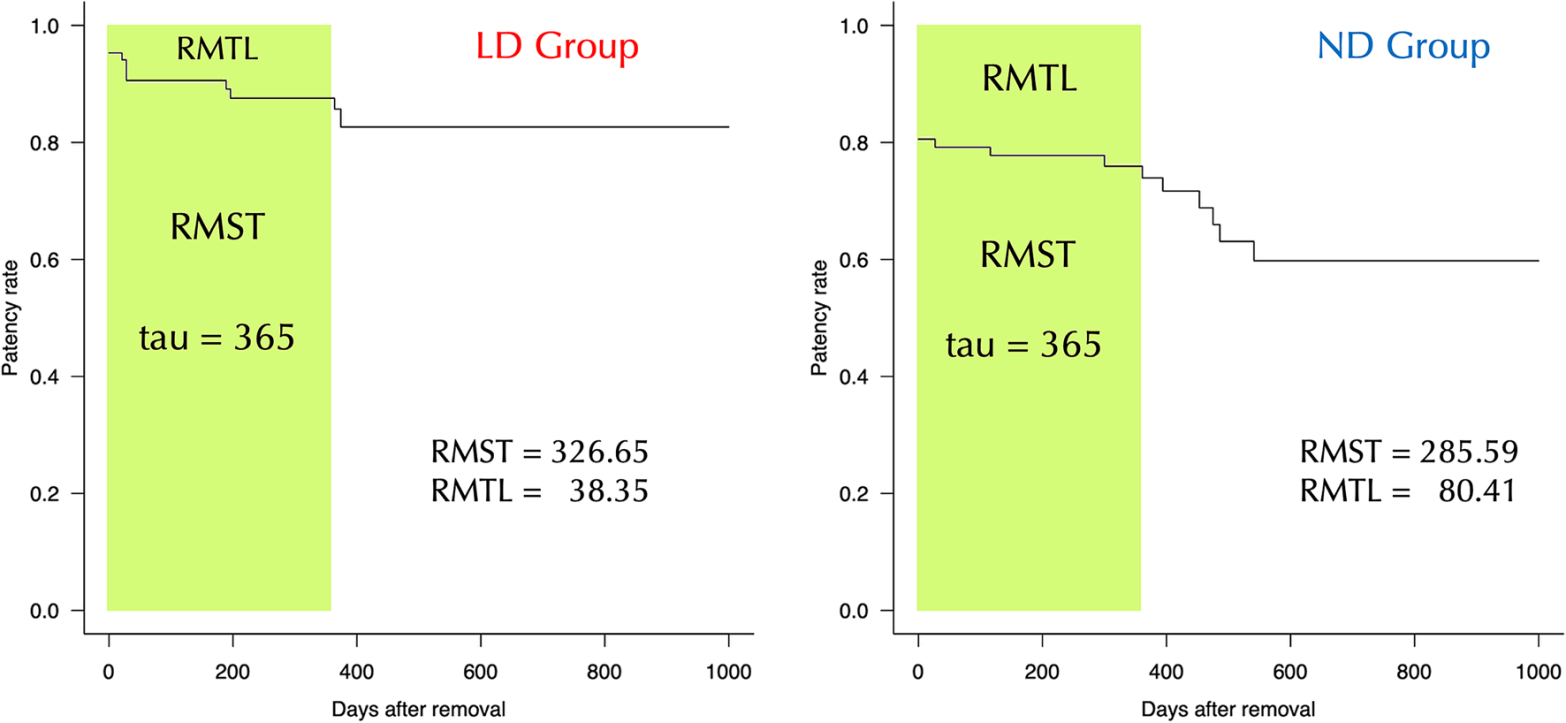
Comparison of survival rates between the two groups at 1 year after tube removal by the RMST method. RMST and RMTL are the areas under and over the Kaplan–Meier survival curve, respectively. The RMST and RMTL in the LD and ND groups with τ = 365 days were 326.65, 284.59, 38.35, and 80.41, respectively. RMST (LD) − (ND) = 42.058 (95% CI: 0.996, 83.121, *p* = 0.045), RMST (LD)/(ND) = 1.148 (95% CI: 0.999, 1.319, *p* = 0.052), and RMTL (LD)/(ND) = 0.477 (95% CI: 0.230, 0.988, *p* = 0.046). The *y*-axis denotes the patency rate, and the *x*-axis shows the number of days after tube removal. Abbreviation: RMST, restricted mean survival time; RMTL, restricted mean time lost.

Figure 4 shows the Kaplan–Meier curve of lacrimal duct patency at 1 year after tube removal. The patency rate of the lacrimal duct was 85.7% (95% CI: 75.4, 91.9) in the LD group and 73.9% (95% CI: 61.7, 82.8) in the ND group. There were a total of 11 recurrences and 23 censored cases in the LD group, whereas there were 18 recurrences and 14 censorings in the ND group at 1 year after tube removal. The RMST with τ = 365 days in the LD and ND groups was 326.65 and 284.59, respectively. In addition, the restricted mean time lost (RMTL) was 38.35 and 80.41, respectively. Results showed that RMST (LD)–(ND) = 42.058 (95% CI: 0.996, 83.121, *p* = 0.045), RMST (LD)/(ND) = 1.148 (95% CI: 0.999, 1.319, *p* = 0.052), and RMTL (LD)/(ND) = 0.477 (95% CI: 0.230, 0.988, *p* = 0.046). Therefore, the patency rate of the LD group was higher than that of the ND group at 1 year after tube removal (Fig. 5).

**Figure 6:**
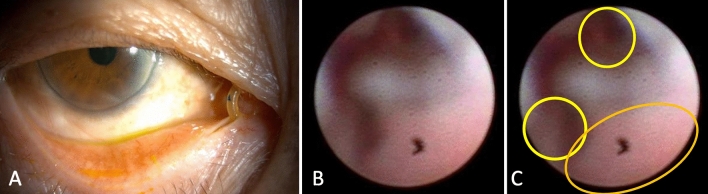
Complications associated with ENDI surgery. (**A**) Punctum slitting. The inferior punctum was stretched nasally caused by the traction of the tube through the nasolacrimal duct. (**B**) Endoscopic image of mucosal granulation in a nasolacrimal duct. Original image of a case complicated with reobstruction during tube placement. (**C**) Annotation for B. The two yellow circles indicate the space where the tubes were placed during the intubation. The orange oval indicates mucosal granulation.

Complications associated with ENDI surgery often involve punctum splitting and granulation on the lacrimal duct mucosa. Mucosal granulations are a significant finding because they can contribute to the recurrence (Fig. 6). 5 cases (5.9%) and 4 cases (5.6%) of punctum splitting were observed in the LD and ND groups, respectively. Meanwhile, 13 cases (15.3%) and 5 cases (5.6%) of mucosal granulations were observed in the LD and ND groups, respectively. The incidence of complications was not significantly different between the groups (*p* = 0.93 and 0.12, respectively).

## Discussion

The first-line treatment for PANDO is DCR. In general, the long-term therapeutic outcomes of ENDI are not considered to be equivalent to DCR. Nevertheless, shreds of accumulated evidence indicate that the results of ENDI are nearly as effective as DCR for canaliculus obstruction and some cases of PANDO (in cases of noninflammatory or partial obstruction)^16–18,33–35^. However, patients with prolonged preoperative occlusions, extended length of postsaccal obstruction, or a history of dacryocystitis tend to relapse after ENDI surgey^17,23,34–36^. The ENDI success rate declines in cases of postsaccal obstruction complicated by chronic dacryocystitis. A longer duration of dacryocystitis leads to more substantial organic changes in the lacrimal duct mucosa, which makes reconstructive surgery with ENDI more complicated^17,34,37^. Therefore, in cases of postsaccal obstruction complicated by dacryocystitis, DCR is recommended.

ENDI operations are generally performed under local anesthesia and are one of the minimally invasive procedures for treating PANDO (Supplementary file, Video 1). As compared with DCR, ENDI has various advantages in terms of surgical time, facial surgical scars, bleeding, and downtime^3,4,11,17,18,23,24,34,38^. In addition, because of the minimal risk of bleeding, ENDI can also be performed in patients receiving systemic anticoagulation and antiplatelet therapy. Hence, further studies are required to compare the long-term treatment effects of DCR and ENDI in patients with PANDO in terms of their pathological conditions (e.g., site, cause, and duration of obstruction).

There have been limited studies on the contribution of tube caliber to the prognosis of PANDO treatment. Several reports have introduced attempts to enlarge the lacrimal ducts using tube stents to prevent restenosis after tube removal. For example, double 0.64-mm Crawford tubes were inserted into one canaliculus^10,39,40^. These studies reported a higher patency rate after canaliculoplasty surgeries using double Crawford tubes. Another study reported that almost similar therapeutic effects were achieved with double silicone intubation using a 0.64-mm Crawford tube and a single wide-diameter 0.94-mm Crawford tube^26^. Our current study demonstrated that the patency rate was significantly higher with the 1.5-mm NST compared with the standard 1.0-mm NST at 1 year after ENDI surgery for postsaccal obstruction.

RMST is a statistical method that has been used for nearly 10 years to evaluate Kaplan–Meier survival curves. The methodology of RMST is to compare the areas under the survival curves to a specific time-point (τ) in the survival curve^31^. RMST is effective when the proportional hazard model is invalid, such as when survival curves are crossed. In addition, because τ can be determined arbitrarily, RMST is useful for comparing the effects of interventions whose follow-up periods do not coincide^41,42^. The 1.5-mm diameter NST was developed and applied clinically in recent years. Consequently, the period for determining the outcomes became shorter than that of the conventionally used 1.0-mm NST. The RMST method was then applied to compare the treatment efficacy between the two groups, because the proportional hazard property was not sustained until the end. In the current study, the τ of RMST was determined as 365 days, considering the following conditions. (1) Clinically, recurrence was mostly observed within 1 year after tube removal. (2) In our institution, postoperative follow-up was usually completed 1 year after tube removal when recurrence was not detected. (3) Due to various factors, such as completion of follow-up or transfer to other medical facilities, the ratio of censoring in the Kaplan–Meier curve increases after 1 year of tube removal. Results showed that the patency rate of the LD group was significantly higher at 1 year after removal, suggesting that larger-diameter tubes had more satisfactory outcomes for the treatment of postsaccal obstruction.

In addition, when comparing the preconditions between the two groups, the ND group had a significantly longer preoperative obstruction period and duration of tube intubation. Previous studies reported that the preoperative obstruction period conferred a risk for recurrence after ENDI surgery^35^. Therefore, we performed a logistic regression analysis to assess the correlation of these factors to recurrence. We found no significant correlation between the preoperative obstruction period and recurrence (*p* = 0.69); however, duration of tube intubation was significantly correlated with the recurrence (*p* = 0.006) in our cohort. This may be because patients with a tendency to relapse tended to have tubes intubated for a longer period of time. The current policy is to perform DCR without more extended tube placement for patients who have a tendency to relapse.

Several limitations are included in this study. This was a retrospective observational cohort study with certain selection biases. The significant extended period of preoperative obstruction and the prolonged duration of tube intubation in the ND group certainly contributed to the sampling biases. Furthermore, since the deployment of the 1.5-mm tubes in August 2017, we adopted our indication of inserting a 1.5-mm NST for all postsaccal obstruction cases (Fig. 3). However, there were cases in which 1.0-mm tubes were inserted for postsaccal obstruction even after deploying 1.5-mm tubes. For example, 1.0-mm tubes were inserted when the patient complained of severe pain during intraoperative endoscopic manipulation and intubating with 1.5-mm tubes was considered challenging. These cases could contribute to sampling biases. Furthermore, not all patients with postsaccal obstruction were treated by ENDI surgeries. For example, we selected DCR over ENDI for patients with a history of acute dacryocystitis, lacrimal duct obstruction for longer than 3 years, or full-length postsaccal obstruction. Moreover, since the decision to perform ENDI or DCR surgery primarily followed the patient’s choice, these selections could also appear to be the factors in the formation of sampling biases.

Next, in terms of lead-time biases, the following points can be considered. First, because 1.5-mm NST was developed more recently, the LD group had a relatively shorter postoperative follow-up period than the ND group did. This constitutes a lead-time bias because the data collection periods do not coincide in the ND and LD groups. Second, we launched the lacrimal duct surgery department at our facility 15 years ago. Over this period, the number of cases operated at our facility has changed both qualitatively and quantitatively. In short, there were more severe cases and fewer surgeries in the beginning, but the number of surgeries and number of patients with milder cases has increased to date. Third, the improvement in the surgeons’ skills over time and the increased probability of successful operations could have contributed to the formation of the lead-time bias. Therefore, to evaluate whether a larger-diameter tube intubation is more effective for postsaccal obstruction, conducting randomized controlled studies with a more significant number of patients in a multicenter study is required.

In conclusion, to the best of our knowledge, this is the first report to compare the postoperative outcomes of bicanalicular silicone tube intubation according to the difference in the tube caliber for the treatment of postsaccal obstruction. The 1.5-mm tube brought significantly less recurrence during the tube placement than the conventional 1.0-mm NST did, which critically increased the patency rate of the LD group 1 year after removal. Although this retrospective cohort study might include various possible limitations, the present assessment elicited data implying the usefulness of 1.5-mm-diameter tube intubation for postsaccal obstruction.

## Supplementary Information

Supplementary Information 1.

Supplementary Video 1.

## Data Availability

The data sets analyzed during the current study are available from the corresponding author (JN) on reasonable requests.

## Abbreviations

CI: Confidence interval
CT-DCG: Computed tomography digital subtraction dacryocystography
DCR: Dacryocystorhinostomy
ENDI: Endoscopic-assisted nasolacrimal duct intubation
NST: Nunchaku-style tube
OR: Odds ratio
SD: Standard deviation
PANDO: Primary acquired nasolacrimal duct obstruction
RMST: Restricted mean survival time
RMTL: Restricted mean time lost
