# Supplementary material for: Comparison of the efficacies of 1.0 and 1.5 mm silicone tubes for the treatment of nasolacrimal duct obstruction
Source: Sci Rep. 2022 Jul 11;12:11785. doi: 10.1038/s41598-022-16018-4 (PMC9276691; doi:10.1038/s41598-022-16018-4)
Supplement: Supplementary file 1 — Supplementary Information 1. [file 41598_2022_16018_MOESM1_ESM.pdf]

Supplementary Figures

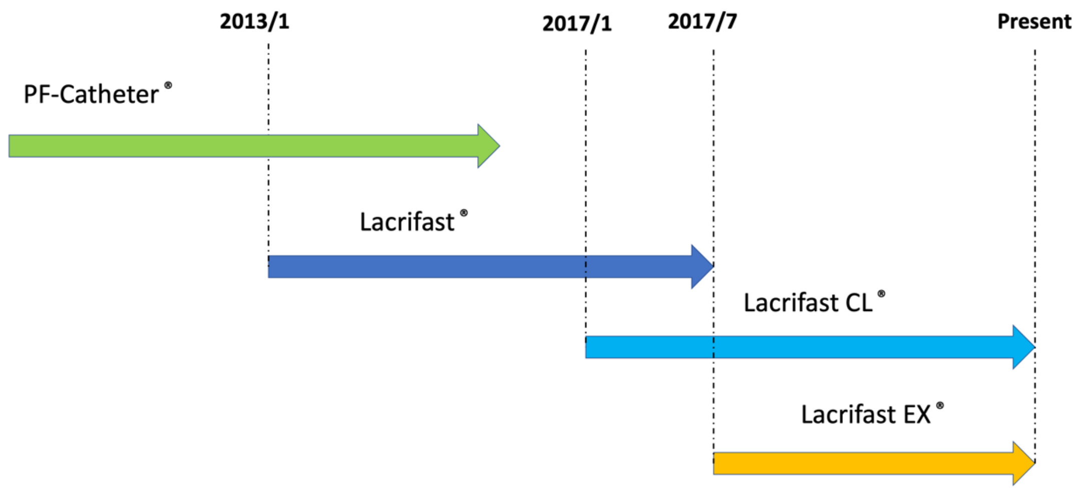

Supplementary Figure 1. Types and periods of tube stents used in our facility

Since the deployment of the 1.5-mm tubes in August 2017, we have adopted our indication of inserting a 1.5-mm NST for all postsaccal obstruction cases.

PF-Catheter; Toray Industries Inc., Tokyo, Japan

Lacrifast, Lacrifast CL, Lacrifast EX; Kaneka Co., Ltd., Osaka, Japan

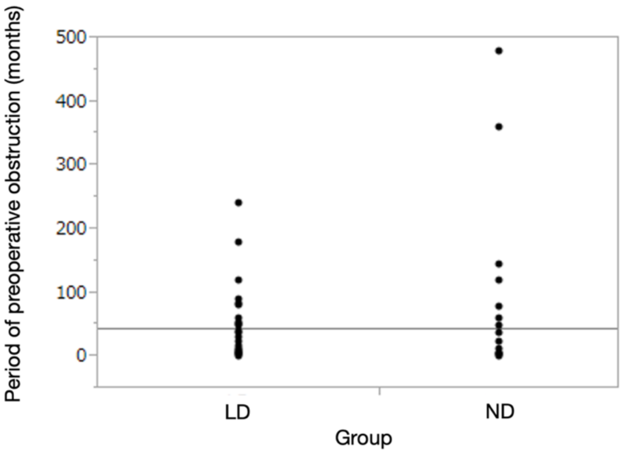

Supplementary Figure 2. Preoperative obstruction period plot

The period of obstruction was denoted based on the duration of chronic epiphora symptoms as described in the patient questionnaire. The period of preoperative obstruction was significantly longer in the ND group ($p = 0.009$).

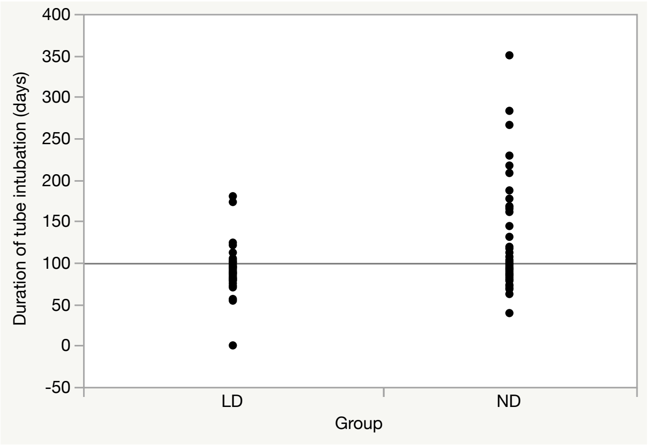

Supplementary Figure 3. Duration of tube intubation plot

The duration of tube intubation was significantly longer in the ND group ($p = 0.036$).
